# Fabrication on the microscale: a two-photon polymerized device for oocyte microinjection

**DOI:** 10.1007/s10815-022-02485-1

**Published:** 2022-05-12

**Authors:** Suliman H. Yagoub, Jeremy G. Thompson, Antony Orth, Kishan Dholakia, Brant C. Gibson, Kylie R. Dunning

**Affiliations:** 1 Australian Research Council (ARC) Centre of Excellence for Nanoscale BioPhotonics (CNBP), Adelaide, South Australia 5000 Australia; 2 University of Adelaide, Robinson Research Institute, School of Biomedicine, Adelaide, South Australia 5005 Australia; 3 Institute for Photonics and Advanced Sensing (IPAS), University of Adelaide, Adelaide, South Australia 5000 Australia; 4 Fertilis Pty Ltd., Adelaide, South Australia 5005 Australia; 5 National Research Council of Canada, Ottawa, ON Canada; 6 School of Physics and Astronomy, University of St Andrews, North Haugh, Scotland KY16 9SS; 7 School of Biological Sciences, The University of Adelaide, Adelaide, SA 5005 Australia; 8 Department of Physics, College of Science, Yonsei University, Seoul, 03722 South Korea; 9 School of Science, RMIT, Melbourne, VIC 3001 Australia

**Keywords:** ICSI, IVF, Infertility, ART, 3D fabrication, High-throughput microinjection

## Abstract

**Purpose:**

Intracytoplasmic sperm injection (ICSI) addresses male sub-fertility by injecting a spermatozoon into the oocyte. This challenging procedure requires the use of dual micromanipulators, with success influenced by inter-operator expertise. We hypothesized that minimizing oocyte handling during ICSI will simplify the procedure. To address this, we designed and fabricated a micrometer scale device that houses the oocyte and requires only one micromanipulator for microinjection.

**Methods:**

The device consisted of 2 components, each of sub-cubic millimeter volume: a *Pod* and a *Garage.* These were fabricated using 2-photon polymerization. Toxicity was evaluated by culturing single-mouse presumptive zygotes (PZs) to the blastocyst stage within a Pod, with several Pods (and embryos) docked in a Garage. The development was compared to standard culture. The level of DNA damage/repair in resultant blastocysts was quantified (γH2A.X immunohistochemistry). To demonstrate the capability to carry out ICSI within the device, PZs were microinjected with 4-μm fluorescent microspheres and cultured to the blastocyst stage. Finally, the device was assessed for oocyte traceability and high-throughput microinjection capabilities and compared to standard microinjection practice using key parameters (pipette setup, holding then injecting oocytes).

**Results:**

Compared to standard culture, embryo culture within Pods and a Garage showed no differences in development to the blastocyst stage or levels of DNA damage in resultant blastocysts. Furthermore, microinjection within our device removes the need for a holding pipette, improves traceability, and facilitates high-throughput microinjection.

**Conclusion:**

This novel device could improve embryo production following ICSI by simplifying the procedure and thus decreasing inter-operator variability.

**Supplementary information:**

The online version contains supplementary material available at 10.1007/s10815-022-02485-1.

## Introduction

Male sub-fertility accounts for 50% of all causes of infertility in couples seeking in vitro fertilization (IVF) treatment [1]. This can be due to low sperm count, aberrant sperm motility, or abnormal morphology [2]. Male sub-fertility is typically addressed using intracytoplasmic sperm injection (ICSI) [3, 4]. This procedure requires an experienced embryologist who uses a micromanipulator to inject a spermatozoon directly into an oocyte [5].

The ICSI procedure requires a microscope equipped with dual manually controlled manipulators, one of which maneuvers a holding pipette and the other an injection pipette [6]. The holding pipette maintains the position of the oocyte using negative pressure during microinjection. The injection pipette is used to aspirate sperm, adjust the orientation of the oocyte, and inject a spermatozoon into the oocyte [7–9]. Adding to the complexity, this process occurs within a confined microliter volume with the embryologist required to consistently refocus and micromanipulate within this miniscule 3D space [10, 11]. With such a manually intensive procedure, it is not surprising that fertilization and implantation rates are positively associated with increased embryologist experience (e.g., those who have performed < 500 ICSI cycles vs those who have performed > 1000 ICSI cycles [12].

Intracytoplasmic sperm injection is routinely performed on multiple oocytes from a single patient [13, 14]. As a result, multiple oocytes are injected sequentially within the same microliter volume of medium. Thus, tracing injected vs non-injected oocytes adds an additional level of difficulty to this procedure. In the instance of many oocytes requiring injection, the procedure may become more inefficient with gametes remaining outside the incubator for an extended period. In turn, this may lead to impaired embryo production [15, 16].

Intracytoplasmic sperm injection can be a technically challenging procedure with its success being influenced by human variability [12, 14, 15]. This procedural variation can induce mechanical stress on the oocyte, negatively impacting fertilization [17]. Additionally, mechanical stress during ICSI may lead to compromised DNA integrity: a cause of oocyte degeneration [18]. Thus, we hypothesized that oocyte microinjection would be simplified with a procedure that requires only one micromanipulator. Furthermore, we hypothesized that a device that houses multiple oocytes in a linear array would decrease the time required for multiple injections and improve tracing of injected vs non-injected oocytes.

In the present study, we designed and fabricated a micrometer scale device that houses the oocyte. The device minimizes oocyte manipulation, requiring only one micromanipulator to perform microinjection. Here, we investigated the use of the device by (1) assessing biocompatibility and embryo culture performance within the device; (2) using the device to microinject presumptive zygotes; and (3) investigate the potential for high-throughput microinjection and improved tracing of injected vs non-injected oocytes.

## Materials and methods

Unless otherwise stated, all chemicals were purchased from Sigma-Aldrich (St. Louis, MO, USA).

### Fabrication of the pod and garage

Our device is comprised of 2 components, the Pod and Garage, which were designed using 3D modeling software (SolidWorks®, Dassault Systèmes SE, Paris, France). Fabrication of the devices was performed using two-photon polymerization technology, using a Nanoscribe Photonic Professional GT printer (Nanoscribe GmBH, Eggenstein-Leopoldshafen, Germany).

The 3D design files (standard tessellation language;.STL) were imported into Describe software (Nanoscribe, Karlsruhe, Germany). The fabrication parameters (i.e., laser printing pattern, structure fill: solid or shell and scaffold) were set into a fabrication job file (.GWL). Fabrication was then performed by direct laser writing. The writing speed and laser power were set to 75 MHz and 75%, respectively.

A 25 × microscope objective, numerical aperture 0.8, was used to focus the laser beam into the sample. The Pods and Garages were fabricated onto a substrate (50 × 50 × 0.55 mm indium-tin oxide glass slide, Fluke Australia Pty Ltd., Baulkham Hills, NSW, Australia). Prior to printing, the glass substrate was rinsed with ethanol then isopropyl alcohol and dried with compressed air.

An IP-S photoresist resin (Nanoscribe GmBH) was used to fabricate the Pods and Garages. Following printing, the excess unfabricated resin was removed by washing in isopropyl alcohol for 8 min, followed by 8-min development (SU-8 developer; Nanoscribe GmBH), and then dried using compressed air. Next, the substrate was submerged in 5% 7X-O-Matic cleaning solution (MP Biomedicals, Solon, OH, USA) overnight at room temperature (RT). Following fabrication, the Pods and Garages were carefully removed from the glass substrate. Once removed, the Pods and Garages were washed three times in 5% 7X-O-Matic cleaning solution at RT. This was followed by three overnight washes in filtered phosphate-buffered saline (PBS; one tablet per 200 mL of Milli-Q water) at RT. The Pods and Garages were stored in PBS at 4 °C until use.

### Animals

All experiments were approved by the University of Adelaide Animal Ethics Committee (M-2019–008) and conducted in accordance with the Australian Code of Practice for the Care and Use of Animals for Scientific Purposes.

Female (pre-pubertal, 3–4 weeks old) and male (6–8 weeks old) CBA × C57BL/6 first filial generation (F1) mice (CBAF1) were obtained from the University of Adelaide Laboratory Animal Services and maintained under 12-h light to 12-h dark cycle with rodent chow and water provided ad libitum.

### Media for gamete and embryo handling and culture

All gamete and embryo culture took place in media overlaid with paraffin oil (Merck Group, Darmstadt, Germany) at 37 °C in a humidified incubator with 5% O_2_ and 6% CO_2_ balanced with N_2_. For the Pod and Garage treatment groups, a single Garage and 3 individual Pods were placed into a drop of culture medium using fine forceps. Culture dishes for all treatment groups were then pre-equilibrated for at least 4 h prior to use. Oocyte and embryo handling were carried out on a heated stage with the temperature set at 37 °C. Mouse tissues were collected in Research Wash Medium (IVF VET Solutions, SA, Australia) supplemented with 4 mg/mL low fatty acid bovine serum albumin (BSA; MP Biomedicals, Albumin NZ™, Auckland, New Zealand). Research Cleave Medium (IVF VET Solutions) was also supplemented with 4 mg/mL BSA and used for embryo culture (manufacturer’s recommended density = 2 μL/embryo). Embryos were cultured from the zygote to blastocyst stage in a single-step culture system (i.e., no media change occurred on day 3 of development).

### Isolation of mouse cumulus oocyte complexes

Pre-pubertal female mice were injected intraperitoneally (i.p.) with 5 IU equine chorionic gonadotrophin (eCG; Folligon; Pacific Vet Pty Ltd., Braeside, VIC, Australia) followed by 5 IU (i.p.) human chorionic gonadotrophin (hCG; Pregnyl; Merck, Kilsyth, VIC, Australia) 46–48 h later. Mice were culled via cervical dislocation, and the ampullae of the oviducts dissected in warmed Research Wash Medium. Ovulated cumulus oocyte complexes (COCs) (14–16 h post-hCG) were isolated by puncturing the ampullae in warmed Research Wash Medium. The isolated COCs were briefly incubated in hyaluronidase (6.1 μM) diluted in warmed Research Wash Medium for 1 min to remove cumulus cells with the aid of gentle pipetting.

### Isolation of mouse presumptive zygotes

Pre-pubertal female mice were injected with eCG (5 IU; i.p.) followed 46–48 h later by 5 IU if hCG (i.p.). Females were then paired overnight with males, with mating confirmed the following morning by the presence of a copulation plug. Female mice were culled via cervical dislocation and the ampullae dissected to isolate presumptive zygotes (PZs) (22–24 h post-hCG). Cumulus-enclosed PZs were incubated in hyaluronidase (6.1 μM) diluted in warmed Research Wash Medium for 1 min to remove cumulus cells with the aid of gentle pipetting.

### Analysis of pod and garage embryo toxicity

The toxicity of the Pod and Garage was assessed using a standard mouse embryo assay (MEA) that used both negative and positive controls, with a certificate of assessment provided (IVF VET Solutions, SA, Australia) [19]. The Pods and Garages were soaked in protein-free MEA medium and incubated overnight at 37 °C in a humidified incubator with 5% O_2_ and 6% CO_2_ balanced with N_2_. Embryo culture drops (10 PZs/20 μL) were then prepared using the MEA medium utilized to wash the Pods and Garages. In addition to the MEA test, embryo culture from the PZ to the blastocyst stage was conducted within Pods docked in a Garage (three PZs docked individually in 3 Pods and a Garage/10 μL) using Research Cleave Medium supplemented with BSA. Fertilization rate was scored 24 h later, with embryos then allowed to develop to the blastocyst stage within Pods and a Garage. At 96 h post fertilization, embryos were considered on-time if at the blastocyst stage (i.e., having a blastocoel cavity ≥ two-thirds the size of the embryo; or expanded; or hatching).

### Analysis of DNA damage/repair in cultured blastocysts (phosphorylated-histone-H2A.X; γH2A.X)

Unless otherwise stated, all immunohistochemistry procedure was carried out at RT. Following either standard embryo culture or embryo culture within the Pods and a Garage, embryos were stained with γH2A.X to assess for double-stranded DNA repair [20]. The blastocysts were fixed in 200 μL 4% paraformaldehyde diluted in PBS (w/v) for 30 min following fixation, blastocysts were washed with PBV (0.3 mg/mL polyvinyl alcohol diluted in PBS) and permeabilized for 30 min in 0.25% (v/v) Triton X-100 in PBS. Blastocysts were then blocked for 1 h with 10% goat serum (v/v; Jackson Immuno, PA, USA) diluted in PBV. Following blocking, blastocysts were incubated overnight in the dark with anti-γH2A.X primary antibody (Cell Signaling Technology, MA, USA) at a 1:200 dilution with 10% goat serum in PBV (v/v). A negative control was included where embryos were incubated in the absence of the primary antibody. Next, embryos were washed three times in PBV before incubation for 2 h in the dark with anti-rabbit Alexa Fluor 594-conjugated secondary antibody (Life Technologies, Carlsbad, CA) at 1:500 dilution with 10% goat serum in PBV (v/v). Embryos were then counterstained with 3 mM of 4′,6-diamidino-2-phenylindole (DAPI; Thermo Fisher Scientific, MA, USA). Finally, embryos were washed three times in PBV and transferred onto a glass microscope slide with DAKO mounting medium (Dako Inc., CA, USA) and enclosed with a coverslip using a spacer (Thermo Fisher Scientific, MA, USA). Embryos were imaged using an Olympus Fluoview 3000 confocal microscope (Olympus Life Science, Tokyo, Japan). Images were captured at 60 × magnification, using the imaging channels Alexa Fluor 594 (red) for γH2A.X (591/614 nm) and DAPI (blue) for DNA (358/461 nm). A z-stack projection for each blastocyst was generated using images captured at 4-μm intervals. The same imaging parameters were kept for each replicate. The intensity of γH2A.X immunostaining was quantified using Fiji ImageJ software (National Institute of Health, MD, USA).

### Comparing standard microinjection vs microinjection within the Pods and a Garage

Microinjection process was assessed to compare technical components for standard microinjection vs within the Pods and a Garage under a micromanipulator. Microinjection was performed in a 60-mm petri dish lid (Falcon, Corning, In Vitro Technologies, VIC, Australia). The microinjection drops were prepared with warmed Research Wash Medium (5 × 10 μL drops at the center of the dish overlaid with paraffin oil). The microinjection dish was pre-equilibrated on a heated stage at 37 °C for at least 4 h before use.

Mouse oocytes were loaded into the microinjection drops to compare standard microinjection (three oocytes/drop) and microinjection within the Pods and a Garage (three oocytes within Pods and a Garage/drop). For standard microinjection, the holding pipette (inner diameter: 17 μm; outer diameter: 80 μm; bevel: 30°; Cook Medical, PA, USA) and injection pipette (inner diameter: 5 μm; outer diameter: 7 μm; bevel: 20°; Cook Medical) were mounted into the micromanipulators. Conversely, for microinjection within the Pods and a Garage, only the injection pipette was used. For standard microinjection and microinjection within a Pod and Garage, the orientation of the oocyte with respect to the polar body was adjusted to either the 6 or 12 o’clock position by placing the injection pipette above the oocyte (in proximity to either the top or the bottom of the oocyte) and moving the pipette along the *z*-axis.

### Microinjection of PZs with fluorescent microspheres

To demonstrate the application of the device for ICSI, microinjection of 4-μm fluorescent microspheres (Invitrogen, Thermo Fisher Scientific, MA, USA) was performed in PZs within the Pods and a Garage. Microinjection of PZs with fluorescent microspheres occurred under a Nikon Eclipse TE2000-E inverse microscope (Nikon Instruments Inc.) equipped with a Tokai Hit ThermoPlate set at 37.5 °C.

Only the injection pipette was loaded into the micromanipulator and used to perform microinjection. The microspheres were aspirated from a separate drop consisting of warmed Research Wash Medium and were then individually injected into each PZ.

Following microsphere microinjection, PZs were transferred from within the Pods and a Garage into pre-equilibrated 2 μL Research Wash Medium on a glass-bottomed confocal dish (Cell E&G, Houston, TX, USA) overlaid with paraffin oil and imaged under the Olympus Fluoview 10i confocal microscope. The fluorescent microspheres were then visualized using the red fluorescence channel (660/680 nm). The microinjected PZs were then cultured in Research Cleave Medium and were allowed to develop to the blastocyst stage.

### Assessment of multiple oocyte microinjection capability within Pods and Garage

The feasibility of microinjecting multiple oocytes within the Pods and a Garage was then tested and compared to microinjecting multiple oocytes using the standard procedure. Microinjection was performed on a microinjection dish with three oocytes loaded per drop for standard microinjection and for microinjection in our device (three oocytes/3 Pods docked in a Garage) within a separate drop. The microinjection drop size for standard microinjection and microinjection within the Pods and a Garage was 10 μL. Under standard microinjection, oocytes were placed into the drop using a pulled glass pipette. For microinjection within the Pods and a Garage, oocytes were loaded into a Pod (1 oocyte per Pod) using a pulled glass pipette. Each Pod was then docked within a Garage using fine forceps.

During standard microinjection, the holding and injection pipettes were utilized to locate and hold individual oocytes prior to microinjection manually. Subsequent microinjection of oocytes was performed after the injected oocyte was released and separated from the non-injected oocyte cohort within the same drop. This process was also performed manually using the holding and injection pipettes. Conversely, for microinjection within the Pods and a Garage, only the injection pipette was utilized. Oocytes were arranged next to each other within our system prior to microinjection. The micromanipulator stage was controlled manually to lead the injection pipette into the non-injected oocyte through the injection pipette channel of the Pod. The micromanipulator stage was then used to facilitate microinjection. Subsequent microinjection of non-injected oocytes was also performed within adjacent oocytes within the remaining Pods docked in a Garage using the micromanipulator stage.

### Comparative analysis of time taken to microinject oocytes within the Pods and Garage vs standard microinjection

Three mouse oocytes were preloaded into individual Pods that were then docked within a Garage. Quantification of time required for individual parameters of the microinjection procedure and comparison between standard and microinjection within the Pods and a Garage was performed. Key parameters considered for this experiment were setting up of pipettes (holding and injection pipettes), holding oocytes before microinjection, and injecting each oocyte. Each parameter within the entire procedure was measured individually (Fig. 5b).

### Statistical analysis

All statistical analyses were performed using GraphPad Prism 8.0 for Windows (GraphPad Software, San Diego, CA). Embryo development data were arcsine transformed prior to statistical analysis. All experimental data were tested for normality to determine whether a parametric or non-parametric test should be used. Statistical analyses were performed using a Student’s *t*-test as described in the figure legends. A *P*-value < 0.05 was considered statistically significant.

## Results

### Design of the Pod and Garage for oocyte microinjection

**Fig. 1:**
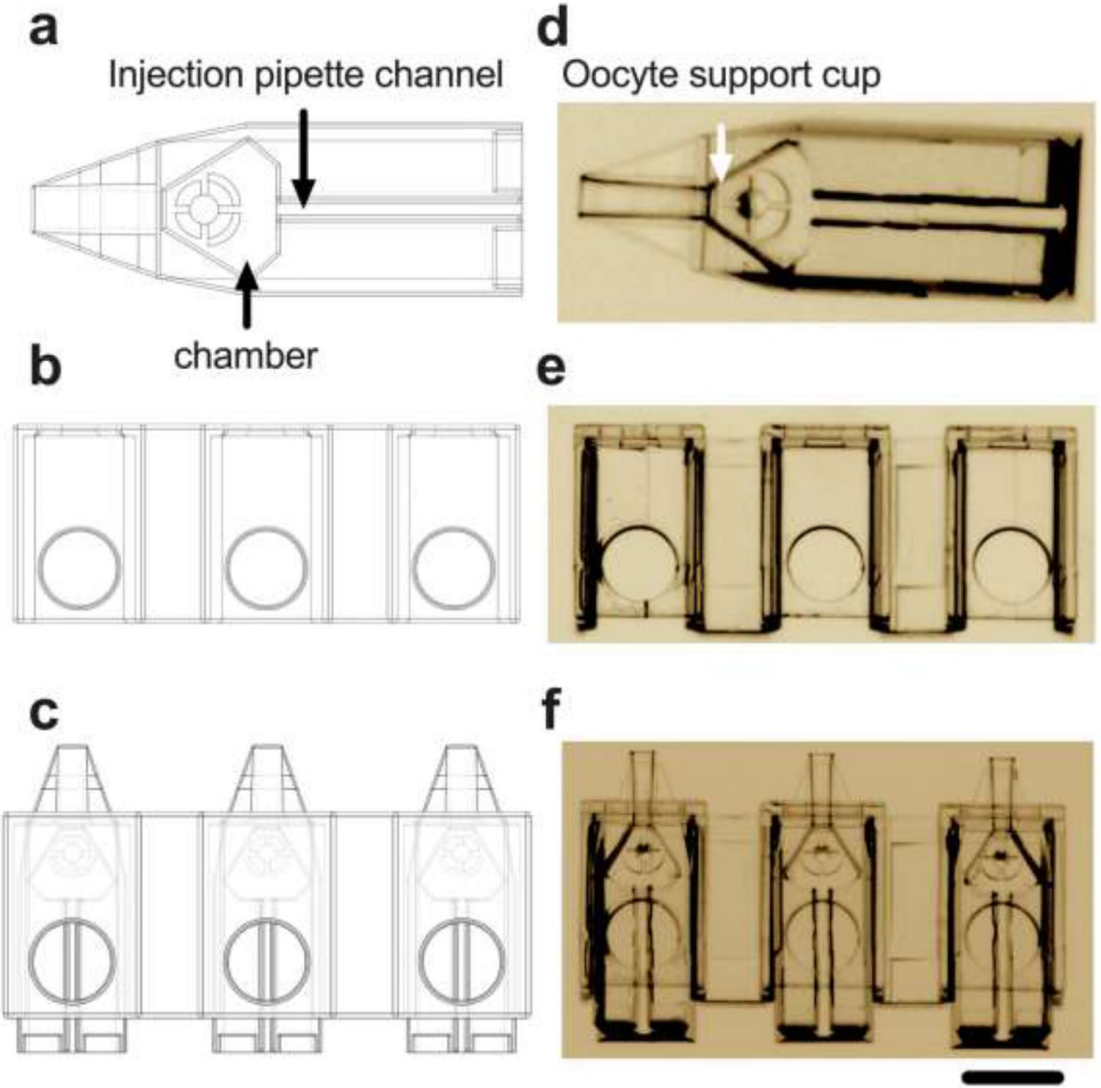
Design and fabrication of a device on the micron scale for oocyte microinjection. Three-dimensional schematic of the Pod (**a**) and Garage (**b**) and an illustration of three Pods docked within a Garage (**c**). The Pod (**d**) and Garage (**e**) were fabricated using two-photon polymerization (Nanoscribe GmBH, Eggenstein-Leopoldshafen, Germany). In (**c**) and (**f**), 3 Pods are docked within a Garage (1500 × 450 × 310 μm; *l* × *w* × *h*). The Pod (725 × 250 × 250 μm) includes access for an injection pipette (*injection pipette channel* (**a** and **d**)) and a chamber with a raised bed that houses and holds the oocyte during microinjection (*oocyte support cup* (**a** and **d**)). Images (**d**, **e**, and **f**) were taken using a 20 × objective with a final magnification of 20 × (Nikon SMZ1500 microscope, Nikon Instruments, Inc., NY, USA). Scale bar = 250 μm

The Pod (Fig. 1a) and Garage (Fig. 1b) were designed using the 3D modeling software SolidWorks®. Required elements of design were (1) to house the mouse oocyte and (2) to enable oocyte traceability by docking multiple Pods within a Garage which simultaneously facilitates high-throughput microinjection (Fig. 1c). Additional design features included access for an injection pipette and a chamber with a raised bed that houses and holds the oocyte during microinjection (Fig. 1a and d). The Garage forms a linear array of sites into which the Pods are inserted horizontally. This device “caps” the Pod, so the oocyte can no longer exit by accident. It also provides the general orientation for the process of ICSI. Two-photon polymerization [21] was used to fabricate the Pod (725 × 250 × 250 μm: *l* × *w* × *h*; Fig. 1d) and Garage (1500 × 450 × 310 μm; Fig. 1e). An image of three Pods docked within a Garage is shown in Fig. 1f.

### Biocompatibility of the Pod and Garage: assessment for potential embryo toxicity

**Fig. 2:**
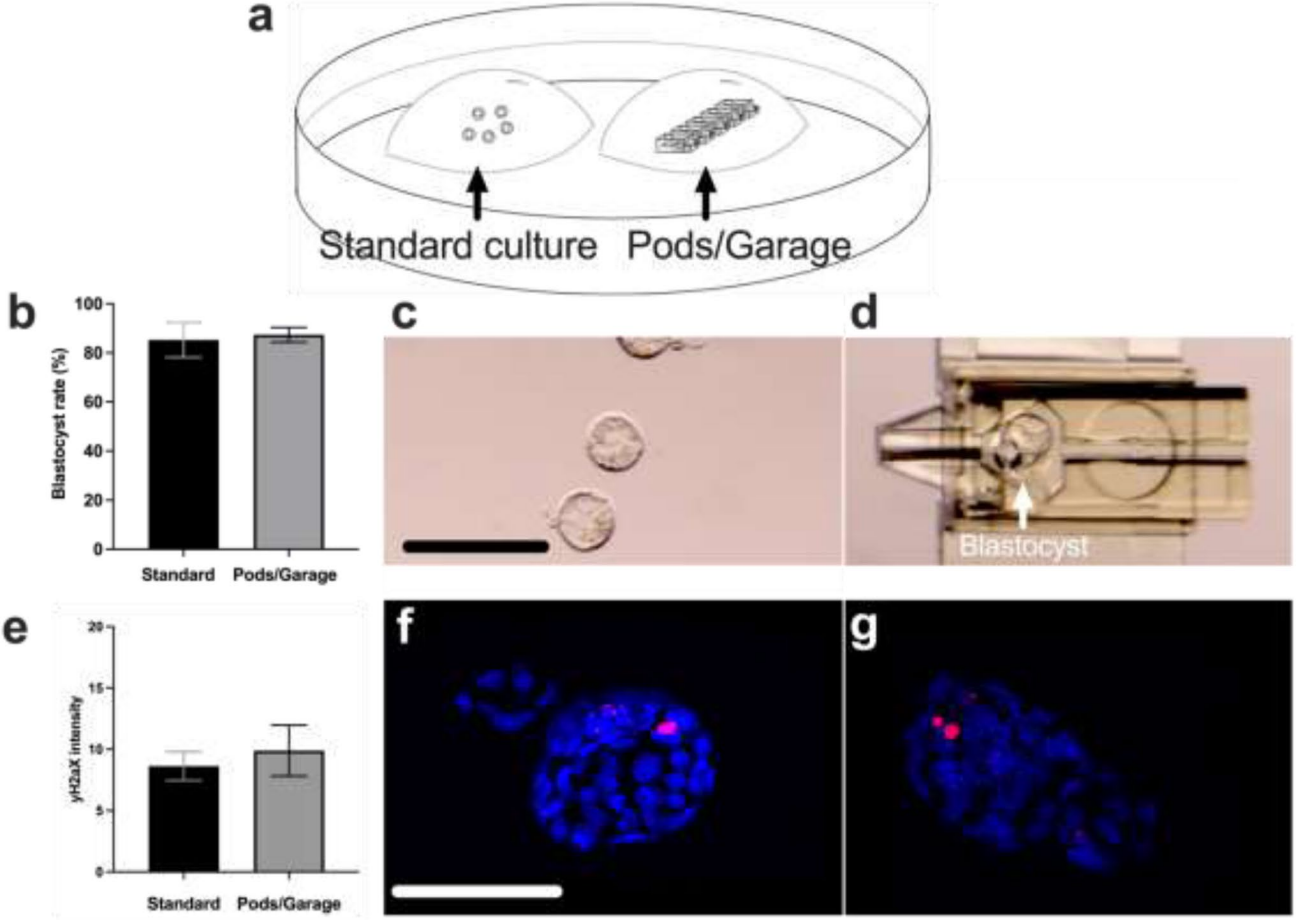
Embryo culture within Pods and a Garage has no impact on preimplantation development or DNA integrity within resultant blastocysts. The experimental design used to assess potential embryo toxicity of the Pod and Garage is shown in (**a**). Mouse presumptive zygotes (PZs) were cultured within a micro-volume of medium overlaid with paraffin oil, with embryo development occurring either on the base of the dish (*standard culture*) or within the Pods and a Garage (*Pods/Garage*) (**a**). Blastocyst rate was calculated from starting number of PZs (**b**). Representative images of blastocysts developed in standard culture or within a Pod and Garage are shown in (**c**) and (**d**), respectively. The integrity of DNA within resultant blastocysts was assessed using γH2A.X immunohistochemistry (**e**). Representative images of DNA integrity within blastocysts cultured in standard culture or within a Pod and Garage are shown in (**f**) and (**g**), respectively [Phospho-histone H2A.X; γH2A.X (*red*)/4′,6-diamidino-2-phenylindole: DAPI (*blue*)]. Images were captured using a 10 × objective with a final magnification of 10 × (Nikon SMZ1500 microscope (**c** and **d**)) or using a 60 × objective with a final magnification of 60 × (Olympus Fluoview 3000 confocal microscope, DAPI: 358/461 nm and γH2A.X: 591/614 nm (**f** and **g**)). All data are presented as mean ± SEM (*n* = 4 experimental replicates, representative of a total of 200–315 embryos for blastocyst development, and a total of 19–33 embryos for DNA integrity). Data for embryo development was arcsine transformed prior to statistical analysis (**b**). All data were analyzed using an unpaired Student’s *t*-test, *P* > 0.05. Scale bar = 200 μm (**c**) and 120 μm (**f**)

We first determined whether the polymer used to fabricate the Pod and Garage was toxic to the mouse preimplantation embryo. Presumptive zygotes were either cultured within a Pod docked in a Garage or on the base of a culture dish, as per standard embryo culture (Fig. 2a). There was no significant difference in development to the blastocyst stage between standard embryo culture and culture within the Pods and a Garage (Fig. 2b–d; *P* > 0.05). Additionally, a commercially sourced MEA test verified the Pods and Garages to be embryo-safe with a blastocyst rate of 89%.

Similarly, when investigating DNA repair within resultant blastocysts, there was no significant difference between embryos cultured using standard conditions compared to those cultured within the Pods and a Garage (Fig. 2e–g; *P* > 0.05).

### Comparison of standard oocyte microinjection and microinjection within a Pod and Garage

**Fig. 3:**
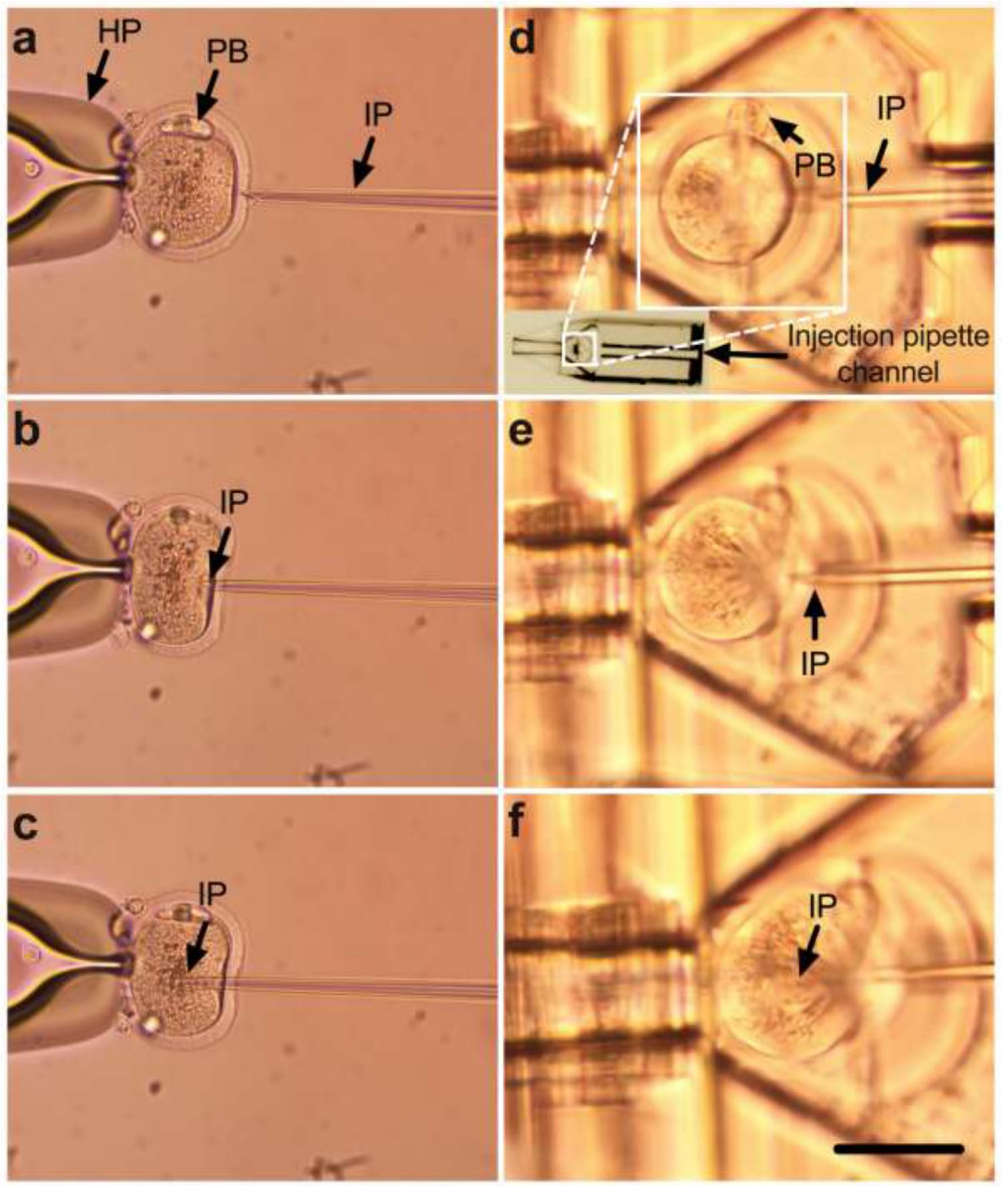
Compared to standard practice, the microinjection of oocytes within a Pod and Garage avoids the need for a holding pipette, simplifying the procedure. Panels show the microinjection of a mouse metaphase II oocyte using either standard microinjection (**a**–**c**) or microinjection within a Pod and a Garage (**d**–**f**). In both instances, the oocyte is oriented such that the polar body (PB) is located at the 12 o’clock position to avoid the metaphase plate during microinjection. During standard microinjection, a holding pipette (HP) is used to hold the oocyte (**a**–**c**). The injection pipette (IP) is pushed against the zona pellucida (**a**), the ooplasm is then penetrated (**b**), and microinjection occurs by the application of negative pressure within the IP (**c**). Oocyte microinjection within a Pod and a Garage is performed using an IP but does not require a HP. The oocyte is loaded into the oocyte support cup (*inset*) which aligns the oocyte with the injection pipette channel (**d**). The IP is pushed against the zona pellucida (**d**), the ooplasm is then penetrated (**e**), and microinjection occurs by application of negative pressure within the IP (**f**). Images were captured using a 20 × objective at a final magnification of 20 × (**a**–**d**) or using a 40 × objective with a final magnification of 40 × (**e**, **f**) (Nikon Eclipse TE2000-E microscope). Scale bar = 120 μm

As we observed no embryo toxicity, we next compared microinjection within Pods and a Garage to standard practice. For standard microinjection, the oocyte was raised from the base of the dish using a holding pipette, which holds the oocyte in place and prevents it from rotating during microinjection (Fig. 3a). Next, the injection pipette was brought adjacent to the oocyte (Fig. 3b). The oocyte membrane was then penetrated by applying a negative pressure through the injection pipette. Only then did microinjection occur (Fig. 3c). Conversely, for oocyte microinjection within our system, the Pod was utilized to hold the oocyte for microinjection (Fig. 3d); thus, there was no need for a holding pipette. Single oocytes were loaded into individual Pods which were then docked into a Garage. The injection pipette was brought next to the oocyte (Fig. 3e). The oocyte membrane was penetrated by applying a negative pressure through the injection pipette (Fig. 3f) during which microinjection occurred (Fig. 3g).

### Microinjection of PZs with fluorescent microspheres within the Pods and Garage

**Fig. 4:**
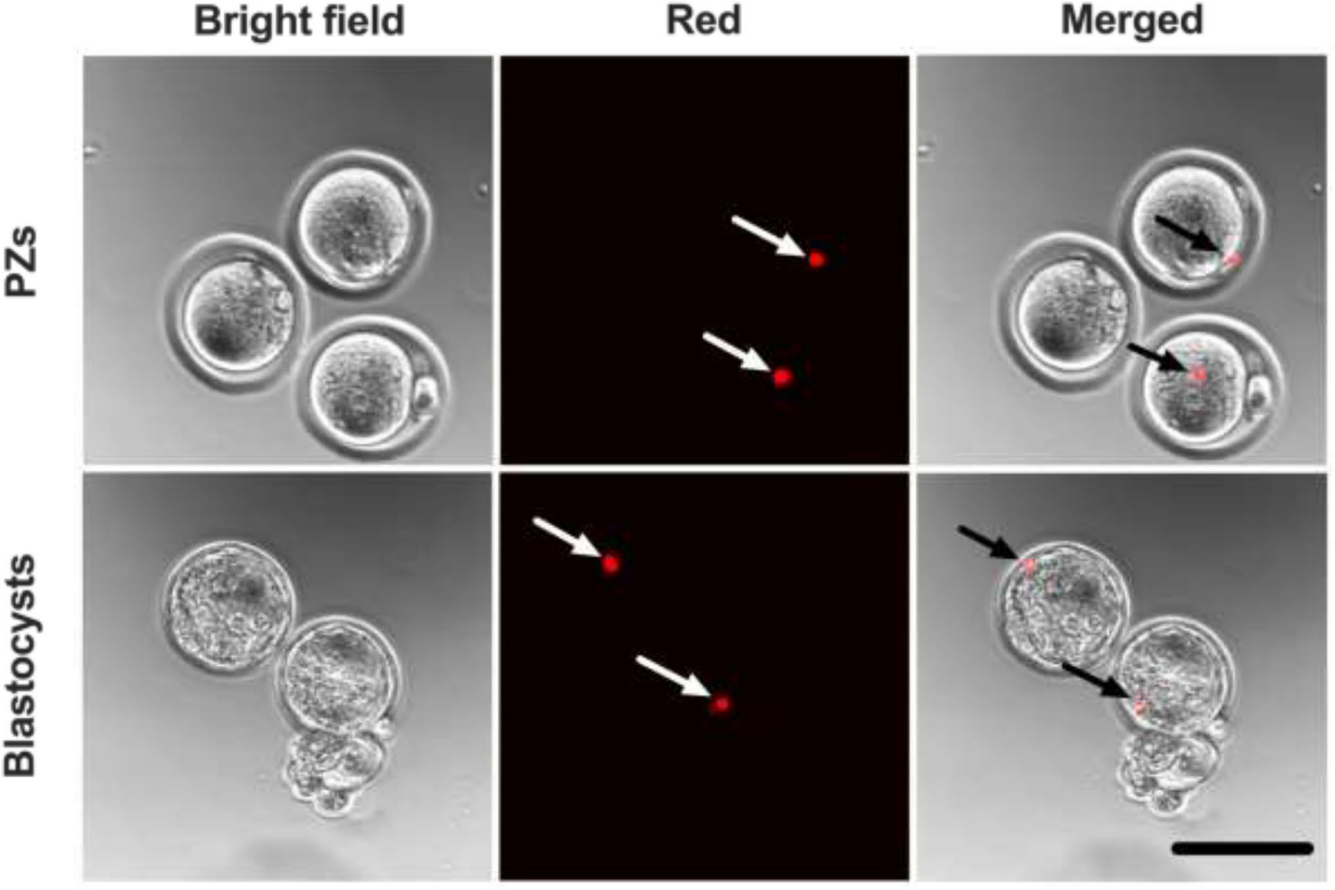
Successful completion of preimplantation embryo development following microinjection of presumptive zygotes (PZs) within Pods and a Garage. To demonstrate the utility of the Pod and Garage system for intracytoplasmic sperm injection (ICSI), mouse PZs were injected with fluorescent microspheres (4 μm) while housed within Pods and a Garage. Following microinjection, PZs were cultured to the blastocyst stage. Images were captured using a 10 × objective with a final magnification of 38 × (Olympus Fluoview 10i confocal microscope). The first column on the left shows PZs under bright field, while the second column shows PZs under the red fluorescence channel (660/680 nm) and the third column shows merged images of the bright field and red channels. Arrows point at the location of fluorescent microspheres within the microinjected PZs and resultant blastocysts. Scale bar = 120 μm

We utilized ICSI injection pipettes that are designed for injection of a human spermatozoon (diameter of head = 4.5 μm) [22]. The injection pipette was not compatible for use with a mouse spermatozoon (diameter of head = 7.9 μm) [23]; thus, ICSI could not be performed using our mouse model. To demonstrate the capability to perform ICSI within our device, we used fluorescent microspheres with a diameter similar to the human sperm head. Fluorescent microspheres (4 ± 0.5 μm) were microinjected into mouse PZs while housed within the Pod and Garage (Fig. 4). Following microinjection, PZs were cultured until the blastocyst stage to assess the impact of microinjection within Pods and a Garage. Embryos successfully developed to the blastocyst stage following microinjection of PZs. This was demonstrated by visualization of fluorescent microspheres within PZs and persistence of these within resultant blastocysts (Fig. 4).

### High-throughput microinjection of oocytes within Pods and a Garage

**Fig. 5:**
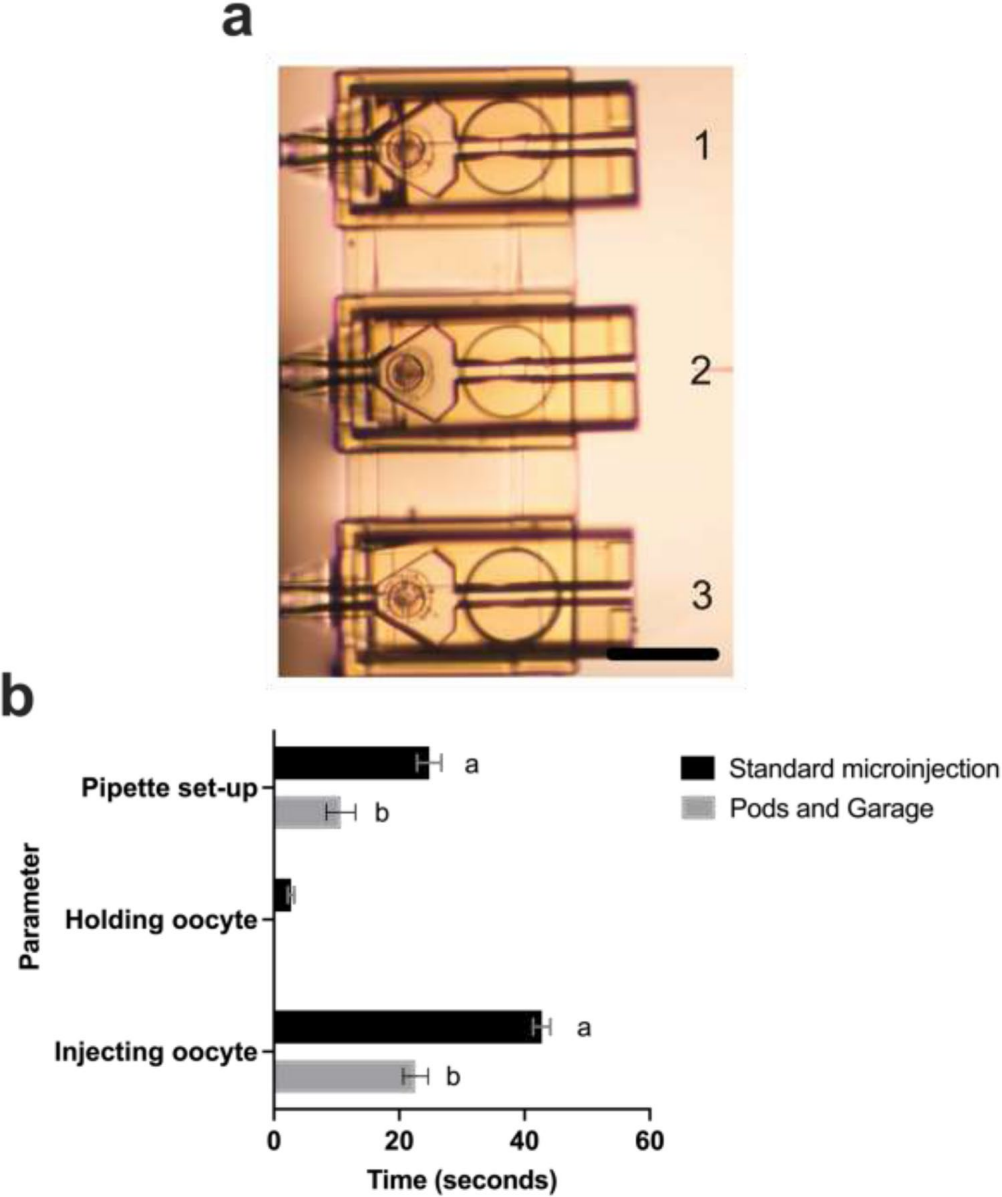
Multiple Pods docked within a Garage improves traceability and enables high-throughput microinjection of oocytes. Three mouse oocytes were preloaded into individual Pods that were then docked into a Garage (**a**). Quantification of time required for individual parameters of the microinjection procedure (pipette setup; holding an oocyte; injecting individual oocytes) and comparison between standard microinjection and microinjection within the Pods and a Garage is shown in (**b**). Images were captured using a 20 × objective with a final magnification of 20 × (Nikon Eclipse TE2000-E microscope). All data are presented as mean ± SEM (pipette setup: *n* = 3–5 experimental replicates; holding an oocyte: *n* = 3 experimental replicates with a total of 45 oocytes for the standard group [N/A for Pod/Garage group]; and injection of individual oocytes: *n* = 3 experimental replicates representative of a total of 18–45 oocytes). Data were analyzed using an unpaired Student’s *t*-test, *P* < 0.01. Different superscripts denote statistical difference between procedures (standard microinjection vs within Pods and a Garage) within a parameter. Scale bar = 200 μm

In standard practice, locating and holding an oocyte prior to microinjection was labor-intensive (Fig. 3). Moreover, when injecting multiple oocytes within the same drop, tracing injected from non-injected oocytes was slow. By housing individual oocytes within single Pods and multiple oocytes/Pods docked within a Garage, traceability of injected vs non-injected oocytes was improved since microinjection occurred in sequence; moving from one Pod to the next (Fig. 5a, 1–3). Furthermore, when compared to standard microinjection, the Pod and Garage system enabled fast high-throughput microinjection (Supplementary Video). Specifically, there was a significant reduction in procedure time for pipette setup (2.3-fold), oocyte holding (2.3-fold) and in the time it takes to inject an individual oocyte (1.9-fold) when microinjection occurred within Pods and a Garage compared to standard microinjection (Fig. 5b; *P* < 0.01).

## Discussion

Successful fertilization following ICSI is dependent on the training and expertise of the embryologist performing the procedure [14, 15]. In the case of a less experienced operator, oocytes may be subjected to higher levels of mechanical stress (causing oocyte lysis) and spend extended periods of time outside an incubator. This negatively impacts fertilization success [24]. Therefore, there exists a need to simplify the procedure and reduce the dependence upon the experience of the operator. We address this need in the current study using our novel device, which we term the Pod and Garage, which minimizes oocyte handling during microinjection. The device improves oocyte microinjection by (1) removing the need for a holding pipette; (2) simplifying and reducing the duration of the procedure; (3) improving the traceability of injected vs non-injected oocytes; and (4) showing the path to high-throughput microinjection.

Following printing of the Pod and Garage using photopolymerization, we verified that the device was suitable for future clinical use. The gold-standard procedure used to determine whether products are suitable for use in the IVF laboratory is to evaluate preimplantation development of mouse embryos under defined conditions and in culture medium that has previously been in contact with the potential toxicant [25]. This test is known as the mouse embryo assay (MEA). It is the primary quality control measure for all equipment and consumables in an IVF clinic [26]. The Pods and Garage passed a standard MEA with an accepted blastocyst rate above 80% [19]. Additionally, embryos cultured within the device developed to the blastocyst stage at rate comparable to those cultured in standard conditions. We also investigated more subtle effects on embryo health. We found similar levels of DNA damage/repair in embryos cultured in standard conditions compared to those cultured within our device. Taken together, these results suggest that the printed device is embryo-safe; however, further work demonstrating safety is required prior to clinical use. In a follow-up study currently underway, cell numbers within the divergent cell lineages of the blastocyst as well as implantation rates and fetal health following embryo transfer will be used to assess the safety of the device. The biocompatibility of the Pod and Garage shown in the current study is consistent with previous work. In those studies, the same polymer was used to fabricate devices for cell culture and drug delivery [27, 28].

Microinjection of oocytes within our device removed the need for a holding pipette, requiring only one micromanipulator equipped with an injection pipette. Furthermore, by docking multiple oocytes within our device, we improved traceability—tracking injected from non-injected oocytes—which also facilitated high-throughput microinjection of multiple oocytes. This is in contrast with standard practice where operation of a second micromanipulator is required to hold the oocyte. In such standard practice, tracing of injected from non-injected oocytes can become difficult in a microliter volume of medium. The Pod and Garage brings a step-change to the procedure, simplifying the process and minimizing oocyte handling and mechanical stress during microinjection.

One crucial point is that direct embryo handling was minimized within our device. The only period when oocytes and embryos were directly handled using a pipette was when they were loaded and unloaded from the Pods. The Pods were docked and undocked into a Garage using fine forceps. Moving the docked Garage between dishes and into different drops (e.g., from the culture dish to an ICSI dish) was performed with the aid of fine forceps. This was simpler than repeated handling and transfer using a micropipette. As no further handling of the oocyte or embryo occurred, we anticipate mechanical stress induced by repeated micro-pipetting and handling in standard practice to be obviated with the use of our device. This will be investigated in a future study.

In the current study, we demonstrated capability to perform ICSI using our device. Microinjection of presumptive zygotes with fluorescent microspheres persisted in the embryo throughout development to the blastocyst stage. This demonstrated that our system supports microinjection with no impairment to the embryo following injection. Importantly, future studies will directly compare standard ICSI with ICSI performed within the Pods and Garage using a compatible animal model. These studies are currently underway. Although these results are encouraging, further examination of short- and long-term development is required before the implementation in the clinic.

In conclusion, microinjection within our device minimizes the requirement for an experienced operator for handling and manipulation. This work suggests the Pod and Garage may improve embryo production and that they may form a precursor to automated ICSI.

## Supplementary information

Below is the link to the electronic supplementary material.

Supplementary file1 (MP4 51316 KB)

## Data Availability

All data generated or analyzed during this study are included in this published article and are available from the corresponding author on reasonable request.

## Code availability

N/A.
